# Septic Shock After Suprapubic Catheter Placement in a Patient With Advanced Multiple System Atrophy

**DOI:** 10.7759/cureus.106507

**Published:** 2026-04-06

**Authors:** Marino A Kokolis-Lattanzio, Denise G Lopez, Mary E Dean

**Affiliations:** 1 Medicine, Touro College of Osteopathic Medicine, Middletown, USA; 2 Internal Medicine, Montefiore St. Luke's Cornwall, Newburgh, USA; 3 Primary Care, Touro College of Osteopathic Medicine, Middletown, USA

**Keywords:** dysautonomia-like disorder, emphysematous pyelitis, multidrug-resistant infection, multiple system atrophy (msa), neurogenic bladder dysfunction, postoperative urosepsis, septic shock (ss), suprapubic catheter placement

## Abstract

Multiple system atrophy (MSA) is a progressive neurodegenerative synucleinopathy characterized by autonomic dysfunction, including significant genitourinary involvement that predisposes patients to urinary retention and infection. We present the case of an 81-year-old man with advanced MSA and chronic urinary retention who developed septic shock shortly after elective suprapubic catheter placement. His hospital course was complicated by emphysematous pyelitis, acute kidney injury, and hemodynamic instability requiring vasopressor support. This case highlights the increased risk of severe infectious complications following urologic instrumentation in patients with MSA and underscores the importance of anticipatory perioperative management in this high-risk population.

## Introduction

Multiple system atrophy (MSA), formerly known as Shy-Drager syndrome, is a progressive neurodegenerative disorder classified among the synucleinopathies and characterized by widespread autonomic, cerebellar, and Parkinsonian dysfunction [1]. Autonomic failure is a central feature of the disease and commonly manifests as cardiovascular instability, gastrointestinal dysmotility, and genitourinary dysfunction. In particular, neurogenic bladder dysfunction frequently leads to urinary retention, catheter dependence, and recurrent urinary tract infections [2,3].

Patients with advanced MSA are, therefore, at increased risk for urosepsis, particularly in the setting of urinary tract manipulation. However, while infections are common in this population, the development of rapid postprocedural septic shock, especially in the presence of emphysematous infection, is not well described. Beyond an increased susceptibility to infection, profound autonomic dysfunction may predispose these patients to severe and early hemodynamic decompensation, as impaired baroreflexes and sympathetic responses limit the ability to maintain vascular tone during systemic stress.

We present a case of septic shock occurring shortly after suprapubic catheter placement in a patient with advanced MSA, complicated by emphysematous pyelitis and cystitis. This case highlights the interaction between urologic instrumentation, severe infection, and dysautonomia, emphasizing the need for heightened perioperative vigilance, including appropriate antimicrobial coverage, careful hemodynamic monitoring, and early recognition of clinical deterioration in this high-risk population.

## Case presentation

An 81-year-old man with a history of type 2 diabetes mellitus and advanced MSA was admitted for elective suprapubic catheter placement due to chronic lower urinary tract dysfunction. His condition was characterized by progressive urinary retention secondary to autonomic failure from advanced MSA, with contributory bladder outlet obstruction from benign prostatic hyperplasia, resulting in chronic incomplete bladder emptying.

The patient had a history of recurrent urinary tract infections, including multiple prior episodes of urosepsis, some complicated by acute kidney injury. He previously required intermittent catheterization but later transitioned to an indwelling Foley catheter due to worsening retention. Given ongoing infections and catheter-related complications, a decision was made to proceed with suprapubic catheter placement, after which he was admitted for close postprocedural monitoring.

Preadmission urine cultures were positive for *Escherichia coli*, and he was started on empiric antimicrobial therapy by the infectious disease team. Due to a history of multidrug-resistant organisms, including vancomycin-resistant Enterococcus and extended-spectrum beta-lactamase-producing bacteria, he received periprocedural prophylaxis with piperacillin-tazobactam and micafungin.

Within several hours postoperatively, the patient developed leukocytosis, with a white blood cell count of 27.25 × 10³/µL, and acute hypotension, with blood pressure decreasing from 167/97 to 94/60 mmHg. Serum lactate was elevated at 2.3 mmol/L and improved to 1.5 mmol/L following fluid resuscitation. Despite an initial response to intravenous fluids, the patient developed persistent hypotension requiring transfer to the intensive care unit and initiation of vasopressor support with norepinephrine (2-50 µg/minute, titrated to maintain a mean arterial pressure of 60-70 mmHg) and midodrine (5 mg orally every eight hours). Vasopressor support was successfully weaned within several hours following hemodynamic stabilization, although the patient remained on midodrine.

**Figure 1:**
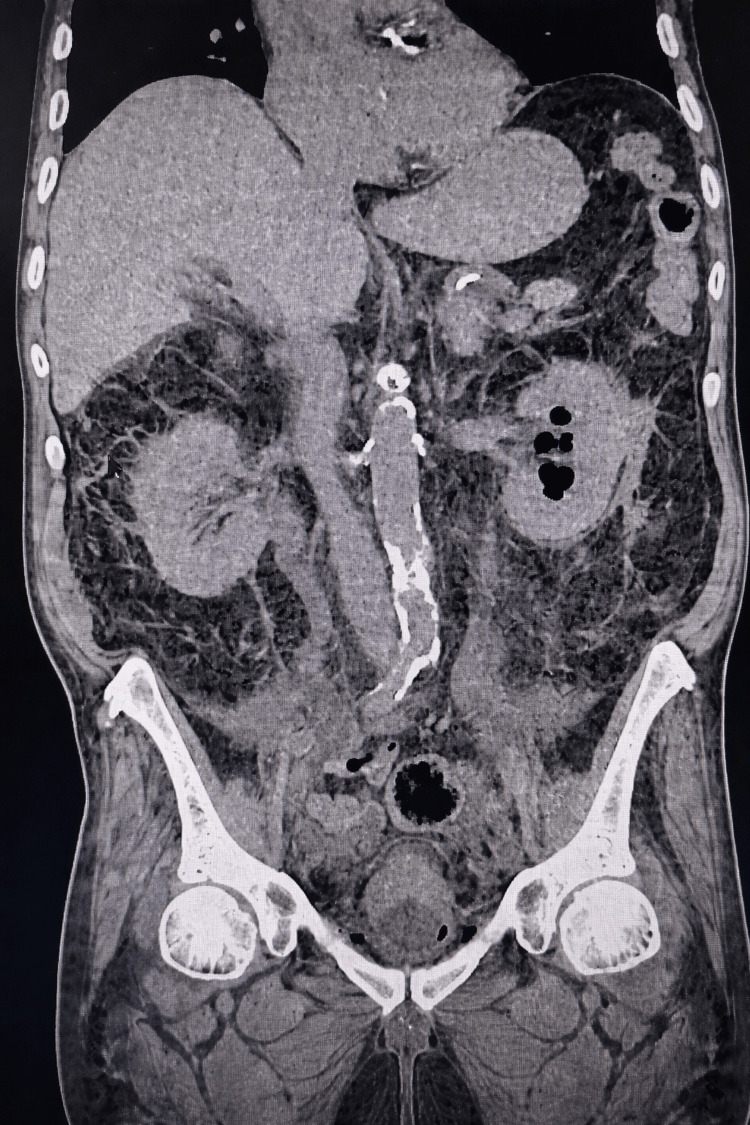
Emphysematous pyelitis and cystitis on CT abdomen and pelvis Contrast-enhanced CT of the abdomen and pelvis, demonstrating findings consistent with emphysematous pyelitis and cystitis, characterized by the presence of gas within the left renal collecting system and along the bladder wall. No evidence of bladder or ureteral perforation is identified CT: computed tomography

Computed tomography of the abdomen and pelvis demonstrated emphysematous pyelitis and cystitis, with air present in the left renal collecting system and bladder wall (Figure 1). No evidence of bladder or ureteral perforation was identified. These findings were attributed to infection rather than procedural complication. Given his history of chronic colonization with multidrug-resistant organisms, the clinical picture was consistent with an infectious exacerbation triggered by urinary tract instrumentation.

**Figure 2:**
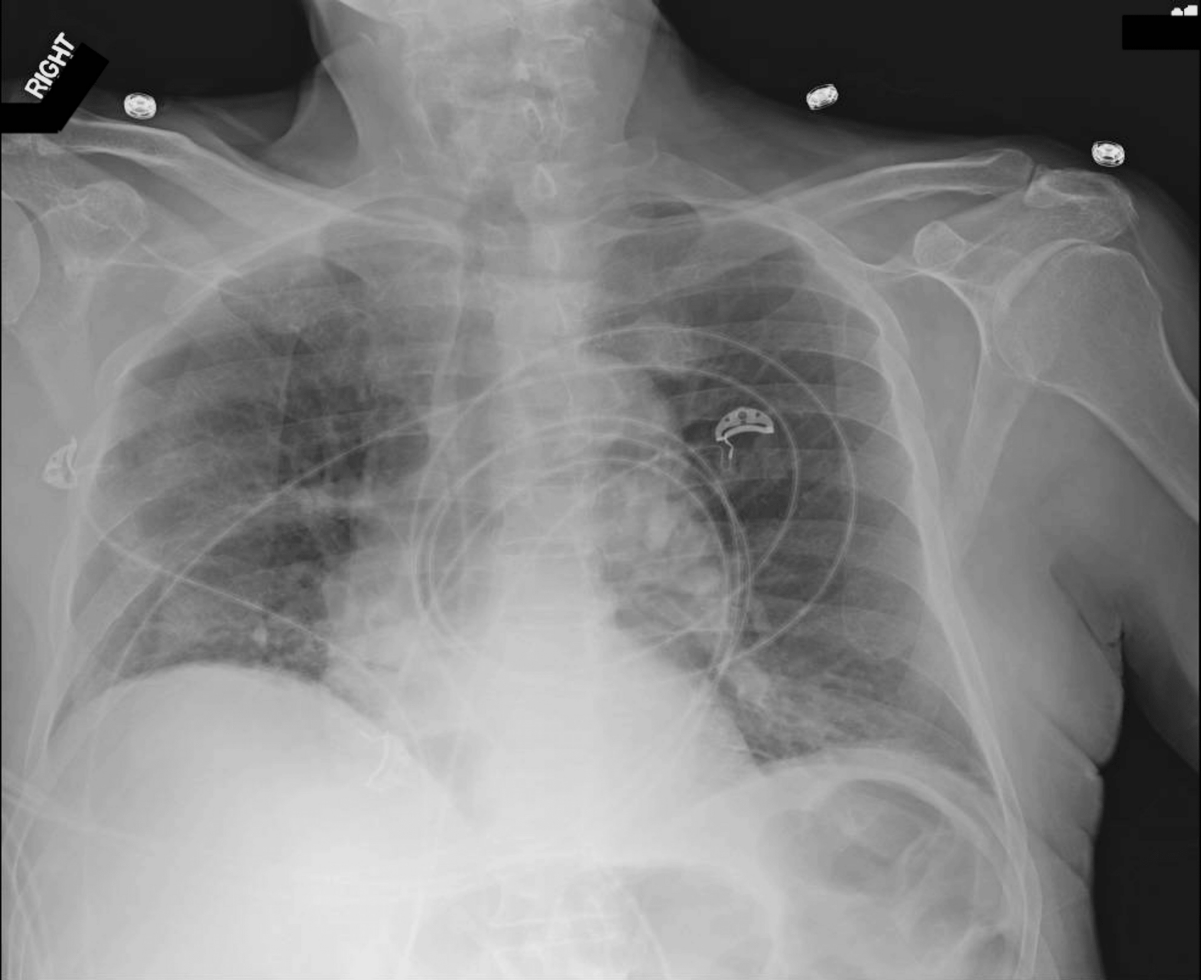
Noncardiogenic pulmonary edema on chest radiograph Chest radiograph demonstrating bilateral pulmonary infiltrates, consistent with noncardiogenic pulmonary edema in the setting of sepsis-related capillary leak

Chest imaging revealed bilateral pulmonary infiltrates, interpreted as noncardiogenic pulmonary edema secondary to sepsis-related capillary leak (Figure 2). Empiric antimicrobial therapy with meropenem (dose adjusted for renal function) and micafungin (100 mg intravenously every 24 hours) was administered for four days and subsequently discontinued following urine culture results demonstrating gram-positive cocci.

The patient developed acute kidney injury on chronic kidney disease stage 3a, with serum creatinine rising from a baseline of 1.07-2.89 mg/dL (estimated glomerular filtration rate (eGFR) 21 mL/minute/1.73 m²). Nephrology consultation attributed this to sepsis-related hypoperfusion in the setting of chronic obstructive uropathy.

Urine cultures obtained following initiation of antimicrobial therapy demonstrated low colony counts (1,000 colony-forming units/mL) of gram-positive cocci. However, prior broad-spectrum antibiotic exposure limited full speciation and sensitivity analysis, reducing diagnostic yield. Based on the clinical presentation and microbiologic findings, the infectious disease team recommended narrowing antimicrobial therapy, with continuation of linezolid (600 mg intravenously every 12 hours) for a total of seven days.

With continued hemodynamic support and targeted antimicrobial therapy, the patient’s clinical status improved. Leukocytosis resolved, with normalization of the white blood cell count to 8.05 × 10³/µL by discharge. Renal function improved toward baseline, with serum creatinine decreasing to 1.66 mg/dL (eGFR 39 mL/minute/1.73 m²). Laboratory findings during hospitalization are summarized in Table 1.

**Table 1:** Laboratory findings on admission and hospital course Summary of laboratory findings during hospitalization, including initial values, peak abnormalities, and values at discharge, with corresponding reference ranges eGFR: estimated glomerular filtration rate; BUN: blood urea nitrogen; CFU: colony-forming unit

Laboratory test	Patient value	Reference range	Interpretation
White blood cell count	27.25 → 24.35 → 8.05 × 10³/µL	4.0-11.0 × 10³/µL	Leukocytosis, resolved
Lactate	2.3 → 1.5 mmol/L	0.5-2.0 mmol/L	Elevated, improved
Creatinine	2.89 → 1.66 mg/dL	0.7-1.3 mg/dL	Elevated, improved
eGFR	21 → 39 mL/minute/1.73 m²	>60 mL/minute/1.73 m²	Reduced renal function
BUN	42 mg/dL	7-20 mg/dL	Elevated
Urine culture	1,000 CFU/mL gram-positive cocci	No growth	Limited interpretation due to antibiotics

## Discussion

This case illustrates the complex interplay between neurodegeneration, autonomic failure, and genitourinary dysfunction in patients with advanced MSA. While urinary dysfunction and recurrent infections are well-described features of MSA, this case highlights the heightened risk of severe septic shock following urologic instrumentation in patients with advanced dysautonomia.

MSA is characterized by the accumulation of misfolded alpha-synuclein within glial cytoplasmic inclusions, leading to widespread neurodegeneration affecting autonomic pathways, the cerebellum, basal ganglia, and spinal motor neurons [4,5]. Autonomic failure in MSA includes impairment of cardiovascular reflexes, which limits the ability to maintain blood pressure during systemic stressors such as infection [1]. As a result, patients may develop profound hypotension that is refractory to standard resuscitative measures.

Genitourinary dysfunction is a hallmark of MSA and often presents early in the disease course. Progressive urinary retention leads to catheter dependence, increasing the risk of recurrent urinary tract infections and urosepsis [2,3]. In this patient, chronic retention was further exacerbated by bladder outlet obstruction, contributing to persistent infection risk. The transition to a suprapubic catheter, while intended to improve drainage and reduce infection frequency, likely served as a trigger for acute infectious exacerbation.

Management of MSA remains largely supportive, with no disease-modifying therapies currently available [6]. Treatment focuses on symptom control, including pharmacologic and nonpharmacologic management of orthostatic hypotension and bladder dysfunction. Pharmacologic management of orthostatic hypotension may include agents such as midodrine and fludrocortisone [7]. In patients with recurrent urinary tract infections, strategies such as optimizing catheter type, minimizing catheter manipulation, and targeted antimicrobial therapy are essential [8].

This case underscores the importance of careful perioperative planning in patients with MSA undergoing urologic procedures. Prophylactic antimicrobial coverage, close hemodynamic monitoring, and early recognition of sepsis are critical. Additionally, clinicians should maintain a high index of suspicion for rapid clinical deterioration due to impaired autonomic compensation. Multidisciplinary care remains essential in the management of patients with advanced MSA [9].

## Conclusions

This case highlights the increased risk of septic shock in patients with advanced MSA undergoing urologic instrumentation. Autonomic dysfunction contributes both to urinary retention and impaired hemodynamic response during infection. Early recognition, appropriate antimicrobial strategies, and close perioperative monitoring are essential to improving outcomes. Multidisciplinary management remains critical in this high-risk population.
